# Adapting IAPT services to support frontline NHS staff during the Covid-19 pandemic: the Homerton Covid Psychological Support (HCPS) pathway

**DOI:** 10.1017/S1754470X20000148

**Published:** 2020-04-28

**Authors:** C.L. Cole, S. Waterman, J. Stott, R. Saunders, J.E.J. Buckman, S. Pilling, J. Wheatley

**Affiliations:** 1 Centre for Outcomes Research and Effectiveness (CORE), University College London – Research Department of Clinical, Educational and Health Psychology, Gower Street, London, UK; 2 Talk Changes (City & Hackney IAPT), Homerton University Hospital Foundation Trust, London, UK; 3 Department of Psychology, Royal Holloway, University of London, Surrey, UK; 4 iCope – Camden and Islington Psychological Therapies Services, Camden & Islington NHS Foundation Trust, London, UK

**Keywords:** cognitive behavioural therapy, disaster response, healthcare workers, IAPT, pandemic, trauma

## Abstract

The Coronavirus (Covid-19) pandemic is exerting unprecedented pressure on NHS Health and Social Care provisions, with frontline staff, such as those of critical care units, encountering vast practical and emotional challenges on a daily basis. Although staff are being supported through organisational provisions, facilitated by those in leadership roles, the emergence of mental health difficulties or the exacerbation of existing ones amongst these members of staff is a cause for concern. Acknowledging this, academics and healthcare professionals alike are calling for psychological support for frontline staff, which not only addresses distress during the initial phases of the outbreak but also over the months, if not years, that follow. Fortunately, mental health services and psychology professional bodies across the United Kingdom have issued guidance to meet these needs. An attempt has been made to translate these sets of guidance into clinical provisions via the recently established Homerton Covid Psychological Support (HCPS) pathway delivered by Talk Changes (Hackney & City IAPT). This article describes the phased, stepped-care and evidence-based approach that has been adopted by the service to support local frontline NHS staff. We wish to share our service design and pathway of care with other Improving Access to Psychological Therapies (IAPT) services who may also seek to support hospital frontline staff within their associated NHS Trusts and in doing so, lay the foundations of a coordinated response.

**Key learning aims:**

(1) To understand the ways staff can be psychologically and emotionally impacted by working on the frontline of disease outbreaks.
(2) To understand the ways in which IAPT services have previously supported populations exposed to crises.
(3) To learn ways of delivering psychological support and interventions during a pandemic context based on existing guidance and research.

## Introduction

The Covid-19 pandemic is imposing pressure, unmatched since World War Two, on Health and Social Care provisions, with NHS hospital frontline staff of various roles and teams encountering vast practical and emotional challenges. Given this, the BMJ has described Covid-19 as a ‘physical and mental health epidemic’, emphasising that during this ‘period of increased stress and uncertainty it is more important than ever for NHS staff to look after themselves’ (BMJ, 2020; Unadkat and Farquhar, 2020). As stated by the World Health Organisation (WHO), healthcare workers are likely to encounter burn-out, traumatic experiences and may use unhelpful coping strategies that can worsen their mental health. This has been indicated by research emerging from China, which found that frontline staff, especially those involved in the diagnosis and treatment of Covid-19 patients, reported high levels of symptoms consistent with insomnia, depression and anxiety disorders. Based on this, the authors issued the striking claim that ‘every clinician is also a patient’ and declared that ‘special interventions to promote mental wellbeing in health care workers exposed to Covid-19 need to be immediately implemented’ (Lai *et al*., 2020; Perlis, 2020). On the other hand, this viewpoint has been subject to criticism, whereby the difficulties faced by frontline staff are perceived as transient and normal emotional reactions to very difficult circumstances, which are likely to subside over time given various resilience factors (Brooks *et al*., 2016). Although it is likely that this will be the case for some, research from the 2003 SARS outbreak found that around 10% of frontline workers, especially those who worked in high-risk roles or were quarantined, continued to show symptoms of anxiety or post-traumatic stress beyond the initial outbreak in 2006 (Wu *et al*., 2009). Due to these risks to staff mental wellbeing, which are likely to be prolonged beyond the initial peak of Covid-19 cases, the premise that the coordinated response need be ‘a marathon, not a sprint’ is key (WHO, 2020). Therefore, it stands to reason that the psychological provisions for staff must adopt a phased approach as the pandemic unfolds. This has been echoed by the British Psychological Society (BPS), whose guidance outlines key principles with regard to supporting frontline staff across the three phases defined as the ‘preparation’, ‘active’ and ‘recovery’ phases. Each of these pose distinct challenges for frontline staff, requiring various forms of psychological input and care (BPS, 2020). Please see the cited article for a more in-depth description of this.

This article provides a summary of the research-informed guidance provided by experts and professional bodies to support mental health services in delivering psychological support for frontline staff. Furthermore, it seeks to highlight the likely prominent role of Improving Access to Psychological Therapies (IAPT) services in implementing this support, given the effectiveness of the stepped-care model and its adaptability to respond to national or local crises. Finally, an outline is provided of the service delivery framework established by Talk Changes (Hackney & City IAPT) to support frontline Homerton Hospital University Foundation Trust frontline staff, which has become known as: ‘Homerton Covid Psychological Support’ (HCPS).

## Guidance for psychological support for frontline staff

Staff working on the frontline are likely to exhibit resilience and commonly use their own coping strategies. However, for many, coping strategies may become unhelpful or experiences on the frontline may pose a risk for the emergence of mental health difficulties or exacerbate existing ones (McKinley *et al*., 2020; WHO, 2020). Therefore, psychological interventions should aim to address the mental health difficulties that have emerged due to trauma or other distressing experiences on the frontline such as ‘moral injury’. The latter, defined as ‘psychological distress that results from actions, or the lack of them, which violate someone’s moral or ethical code’, has been emphasised and viewed as a priority by experts (Greenberg *et al*., 2020). For healthcare workers, this may include being unable to offer possibly life-saving treatment, such as ventilation, to some patients over others due to a lack of available resources (Dean *et al*., 2019). Through moral injury, frontline staff are likely to develop negative thoughts, concerning themselves or others, accompanied by strong negative emotions (e.g. guilt), which over time can result in mental health difficulties including post-traumatic stress disorder (PTSD). The emergence of such difficulties is likely to be impacted by whether ‘they [staff] are supported, before, during, and after a challenging incident’, reiterating the need for a phased approach (Greenberg *et al*., 2020). Furthermore, as these difficulties may originate from a multitude of potentially traumatising frontline experiences, specialists have stressed the importance of a trauma-informed response across these phases. As summarised by the Covid Trauma Response Working Group, this will be achieved through delivering interventions that acknowledge various ‘dos and don’ts’, which echo the key principles of trauma-informed care (Billings *et al*., 2020; Sweeney and Taggart, 2018).

It is important to note that final year medical students, junior or formerly retired staff and volunteers are expected to be deployed to the frontline by various Trusts. Research strongly suggests that formal or pre-deployment training and experience in healthcare roles serve as protective factors (Brooks *et al*., 2015). Therefore, for these individuals in particular, psychological input may be a ‘lifeline’ when working in these teams and contexts, whilst they adjust to their new roles. Moreover, research suggests that clinical staff exhibit lower psychological resilience, especially if their hours are long, than administration staff (Sull *et al*., 2015). The authors of the study suggest that this is due to a tendency for clinical staff to hesitate in taking up social or formal support. As a result, it is crucial for frontline leadership to promote uptake and for organisational efforts to be made to enhance the presence of psychological services to aid access for those in such clinical roles.

**Figure 1.**
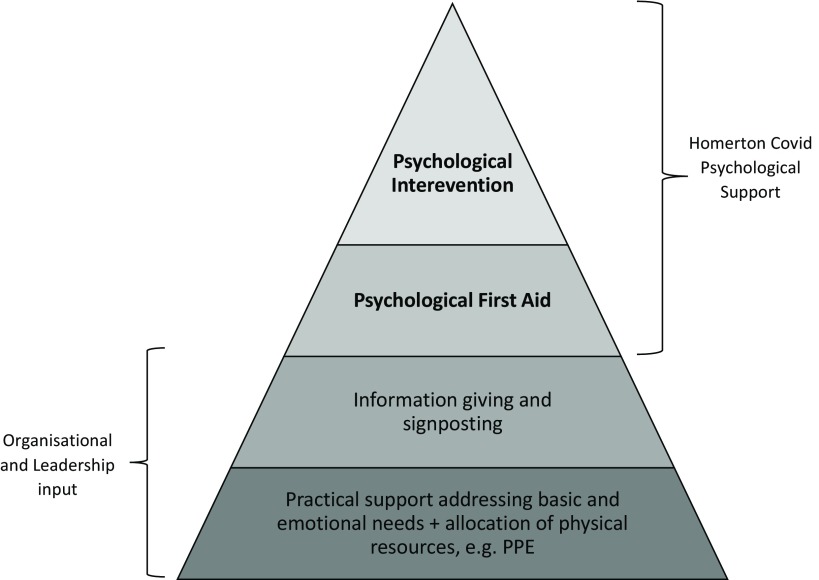
The role of Homerton Covid Psychological Support as part of the stepped psychological response (BPS, 2020). PPE, personal protective equipment.

The time frame and nature of psychological intervention has been debated, with several barriers to delivery highlighted. Drawing on accounts of staff support from China, an article in *The* *Lancet* stated: ‘The implementation of psychological intervention services encountered obstacles, as medical staff were reluctant to participate in the group or individual psychology interventions provided to them. Moreover, individual nurses showed excitability, irritability, unwillingness to rest, and signs of psychological distress, but refused any psychological help and stated that they did not have any problems’ (Chen *et al*., 2020; p. 1). As an alternative to psychological intervention, staff suggested they wanted ‘more rest without interruption and enough protective supplies’. This indicates that a majority of frontline staff were unwilling to engage with psychological support and secondly, that staff were more focused on practical support. This is in line with the theoretical assumptions of Maslow’s hierarchy of needs, which claims that individuals are less likely to address their psychological needs when basic needs remain unmet, including security and safety (Maslow, 1943). However, as always, there should be caution in generalising the contexts of one nation to another, along with the research findings that emerge from them. As such, one reason for the low uptake of psychological intervention amongst Chinese frontline staff may stem from socio-cultural factors; for example, Huang and colleagues (Huang *et al*., 2019) suggest that recognition of mental health problems in China is low compared with other countries such as the UK. Therefore, low recognition of symptoms or stigma amongst Chinese frontline staff may be a reason for the claims reported by *The Lancet* and for this reason, update of psychological intervention amongst frontline staff in the UK context may differ; this is a possible hypothesis for further research. Regardless, it is clear that psychological support, such as our proposed model (HCPS) should not be offered as a standalone or disconnected from other practical forms of support. Instead, it is important that psychological provisions are embedded in a wider framework of support (see Fig. 1) and follows successful attempts to address practical issues through organisational efforts and effective leadership (Brooks *et al*., 2016).

Staff can also acquire psychological support from within frontline teams themselves, as well as less formal sources from their wider systems. Many hospitals in the UK currently have support structures in place for staff impacted by the emotional challenges related to their roles. This includes Schwartz Rounds: an open yet facilitated forum for staff of all clinical and non-clinical roles to reflect on the emotional impact of their work. These have been shown to have positive benefits for the individuals that take part but also for the wider organsiation and its patients (Flanagan *et al*., 2019; Taylor *et al*., 2018). Peer support groups have also been beneficial for the psychological and emotional wellbeing of nurses and doctors alike (Gerada, 2016; Jackson, 2018). In recent years, encouraging compassion amongst healthcare staff has become a target of team-based interventions. For example, ‘Taking Care, Giving Care’ rounds or ‘Compassion Circles’ have proven to be feasible and effective for enhancing staff emotional and psychological wellbeing (Flowers *et al*., 2018). Despite these beneficial team-based support structures, effective supervision and leadership remains as a cornerstone for supporting staff wellbeing (Babin and Boles, 1996). In addition, frontline staff can seek support from non-formal sources situated within their wider systems; for example, during the 2014 Ebola epidemic, staff reported religion, country-wide morale and community or family support as protective factors (Raven *et al*., 2018).

## The role of IAPT services

Since 2008, services under the IAPT programme have offered evidence-based psychological interventions to individuals with common mental health difficulties (anxiety disorders and depression) across England (Clark, 2011). These interventions, which have been predominantly CBT orientated, are delivered in line with guidance from the National Institute for Health and Care Excellence (NICE) that advocates a stepped-care model of service delivery, which has been shown to allow services to see more patients in a manner that is clinically and cost effective (NICE, 2011; Boyd *et al*., 2019). As part of this model, a range of high-intensity interventions are recommended that have expanded beyond CBT to include Interpersonal Therapy (IPT), Couples Therapy, Dynamic Interpersonal Therapy (DIT) and Counselling for Depression (CfD), as well as Eye Movement Desensitisation Re-processing therapy (EMDR) for PTSD (NICE, 2018). Whilst undergoing these treatments, service users self-report their symptoms using psychometric measures that comprise the ‘minimum data set’ (MDS) and anxiety disorder-specific measures (ADSM). A full description of measures and conditions treated under IAPT services can be found via the IAPT manual (National Collaborating Centre for Mental Health, 2019). Through IAPT services, approximately one million individuals per year start treatment, with outcomes England-wide largely resembling those of efficacy studies (NHS Digital, 2020b). However, a significant degree of variability in outcomes exists between services given organisational variables such as wait-times (which can exceed 9 months), treatment dose offered and attendance rates. Furthermore, social deprivation has been found to impact the effectiveness of interventions, meaning that services in areas of high deprivation with possible inadequate funding are likely to experience a detriment in recovery rates (Clark *et al*., 2018; Delgadillo *et al*., 2016).

**Figure 2.**
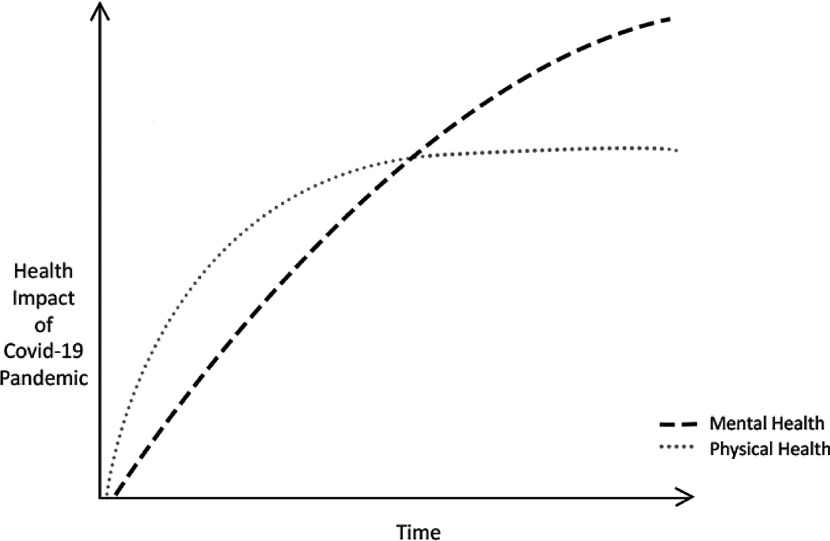
The conceptual ‘unseen curve’ of Covid-19-related mental health problems.

In times of local or national crisis of a natural, man-made or industrial nature, individuals are more likely to develop mental health problems (Makwana, 2019). Although there is no rigorous published data to evidence this in the UK nationwide, it is expected that crises lead to a spike in referrals to mental health services, whilst many affected may not seek help, as observed amongst emergency and frontline workers of the 2005 London Bombings (Brewin *et al*., 2010). Therefore, it is important for services to screen, assess and treat individuals, whilst paying careful attention to the nature of the crisis and the trajectory at which it unfolds, both of which may impact the emergence of mental health problems over time for the populations affected. This has been achieved by services and psychological care pathways that have been designed on or integrated into IAPT services. This includes, but is not limited to, The Ebola Psychological Support Service (EPSS), The Grenfell Health and Wellbeing Service (psychological therapy for communities affected by the Grenfell Tower fire) and the Trauma Outreach, Screen and Support Service for London Terrorist Incidents (Albert, 2019; Bailey and Heke, 2019; Waterman *et al*., 2018). EPSS, which informed the service model described in this paper, sought to psychologically support Sierra Leonean nationals working on the healthcare frontline for non-government organisations (NGOs) during and after the 2014 Ebola epidemic. This consisted of three phases based on the IAPT stepped-care model: (1) screening and psychological first aid via a group format, (2) workshops targeting commonly reported difficulties, and (3) group CBT for anxiety and depression. EPSS, which has now ceased, was found to be effective in reducing mental health difficulties across the three phases. Meanwhile, the Grenfell Health and Wellbeing Service and the Trauma Outreach, Screen and Support Service for London Terrorist Incidents continue to operate, with largely good clinical outcomes reported outside of formalised studies (Albert, 2019; Bailey and Heke, 2019). It is likely that these outcomes lend themselves, in part, to the features of the IAPT model such as supervisory structures, ease of access, stepped-care and the IT systems utlised that permit effective evaluation. Taking this into account, the IAPT model has proven itself in having the adaptability, workforce and existing infrastructures to coordinate a planned, phased and stepped-care approach for the emerging Covid-19 crisis. This approach will need to be sustained beyond the initial peak of the outbreak and throughout what is being described as the ‘unseen curve’: a trajectory of mental health problems inflicted upon healthcare workers beyond their initial distressing and traumatic experiences (Fig. 2). Please note, this figure is solely conceptual.

## Homerton Covid Psychological Support

**Figure 3.**
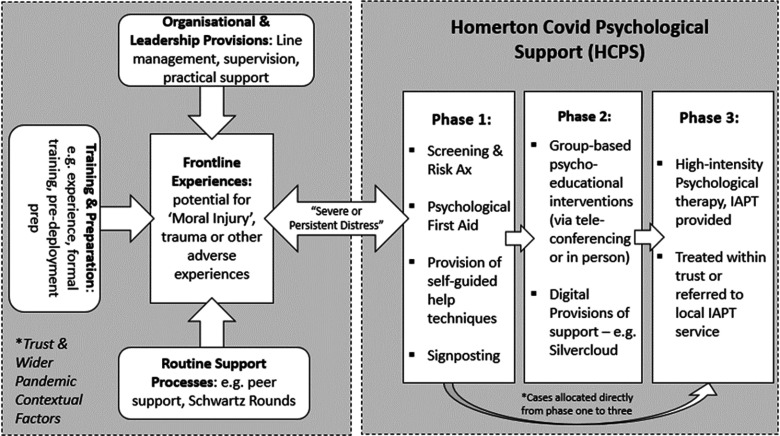
HCPS pathway for frontline staff experiencing mental health difficulties. Some cases are allocated directly from phase 1 to 3 due to having pre-existing mental health problems or severe symptoms.

Taking into account the above guidance, which has largely been informed by research, collectives of experts and professional bodies, we have designed a new care pathway for Homerton Covid-19 frontline staff: Homerton Covid Psychological Support (HCPS; see Fig. 3), which has been approved by local directors. Individuals eligible for this support are defined as ‘frontline workers’ across health and social care provisions of Homerton University Hospital Foundation Trust. This includes, but is not limited to, doctors, nurses, midwives, paramedics, social workers, care workers and volunteers (Cabinet Office, 2020). Support will also be offered to those who uphold the sector without a clinical input such as cleaners, administrators and security personnel. An online portal has been established to receive self- or signposted referrals, which will be promoted within the Trust by management, supervisors and the communications team, as well as through outreach initiatives. These referrals are accepted on the basis of the presence of distress that is deemed ‘*severe or persistent*’ and after internal organisational support efforts made by the Trust have not been adequate in meeting psychological or emotional needs (Greenberg *et al*., 2020). As no routine measures are used to track the psychological wellbeing of frontline staff, distress severity will be subjective to the member of staff and their supervisors. However, upon referral, IAPT standardised measures (the minimum data set, MDS) will be completed by those referred to gauge the degree of distress associated with mental health symptoms, as well as functional impairment (National Collaborating Centre for Mental Health, 2019). Individuals that are below ‘clinical caseness’ will still be offered support as part of phases 1 and 2 (outlined below). If the individual declines HCPS, efforts will be made to signpost them to alternative forms of support that best meet their needs.

HCPS has been designed based on the previously mentioned Ebola Psychological Support Service (EPSS), which was designed and delivered by South London and Maudsley NHS Trust (SLAM). Please read Waterman and colleagues (Waterman *et al*., 2018) for a full description of the three-phased service model. EPSS has accumulated a body of preliminary evidence and feasibility has been indicated (Waterman *et al*., 2018; Waterman *et al*., 2019). Amongst those who took part in all phases of EPSS, improvement was observed on all measures of mental health difficulties included (standardised and bespoke). A group CBT anxiety and depression intervention for staff, resembling a low-intensity IAPT group, was also found to be effective for frontline staff as a standalone (Cole *et al*., 2020). It is important to note that all EPSS interventions and materials were based on those already delivered in IAPT services, i.e. CBT of both low and high intensity. Therefore, we anticipated that the model would easily be adapted to the UK, NHS context. Inspired by the above we have plans to provide the following (Fig. 3), which will most likely operate during the ‘active’ and ‘recovery’ phases of the Covid-19 outbreak (BPS, 2020). We anticipate that these provisions will extend beyond the year that follows the initial peak of the outbreak.

### Phase 1

This is a ‘screening and psychological first aid’ provision offered during the acute or ‘active’ phases of the outbreak (BPS, 2020). Referrals are received via a dedicated online portal. During the sessions conducted remotely, the practitioner will screen for mental health symptoms and conduct a risk assessment. Following this, they will facilitate the caller’s recognition of their own coping strategies and resilience factors but also suggest some additional coping strategies. This will be formalised as a ‘psychological wellbeing plan’, which the frontline staff can implement in a self-guided manner. These are later reviewed by the practitioner. We paid careful attention in ensuring that this phase does not resemble single-session psychological debriefing, which can cause possible harm (Brooks *et al*., 2020). A copy of our phase 1 protocol can be requested via the corresponding author (C.C.).

### Phase 2

Phase 2 consists of CBT-based interventions, which can be facilitated in a group format by a practitioner through video-conference technologies or at Homerton Hospital following social distancing measures. These sessions would cover the same topics as the EPSS: ‘simple coping strategies based on behavioural and cognitive approaches that staff could use as self-help’ (Cole *et al*., 2020; Waterman *et al*., 2018). Difficulties addressed include stress, anxiety, unhelpful coping, bereavement and grief, low mood and sleep problems. It is important to note that peer support was a powerful enabling process for frontline staff engaging with EPSS interventions, strengthening the rationale for the group format outlined above. In addition to these CBT-orientated interventions, HCPS will also be providing the ‘20minCareSpace’ pilot intervention to frontline staff on-site or remotely. Developed by Jones, Bradley and Waites as cited in Scior and Clements (2020), this brief intervention is based on the previously mentioned ‘Compassion Circles’ and has the aim of promoting self-care and self-compassion amongst frontline staff to serve as a buffer against mental health difficulties. It will be facilitated by two psychological practitioners from the HCPS team.

### Phase 3

In Phase 3, a ‘screen and treat’ approach will be adopted, whereby frontline staff who have taken part in phases 1 and 2 and yet have persistent difficulties, will be rapidly assessed and treated with NICE-recommended interventions for mental health difficulties such as PTSD. Some individuals screened during phase 1 who have pre-existing mental health difficulties or are experiencing severe symptoms, as determined by interview and measures, will be directly allocated to phase 3 (Fig. 3). Outreach initiatives will also be conducted to encourage referrals from frontline staff who did not refer during the earlier stages of the outbreak. This is important as it has been recognised that some of the most impacted crisis responders are hesitant to refer for support (Brewin *et al*., 2010). These phase 3 interventions will be provided by IAPT high-intensity practitioners. Overall, this phase shares similarities to the Psychological Trauma Outreach, Screen and Support Service for London Terrorist Incidents (Albert, 2019). Interventions can be delivered either by Talk Changes (City & Hackney IAPT) or by the local service associated with the frontline staff’s residence. Given social-distancing measures, this therapy may continue to be delivered remotely (by telephone or online), although during later phases of the outbreak face-to-face delivery may be possible.

### Supervision structures

Staff will receive routine supervision from a consultant clinical psychologist as well as specialist skills supervision from a consultant clinical psychologist who is part of the expert Covid Trauma Response Working Group. Given the nature of the support the workforce will be providing and due to the risks of secondary trauma, we will ensure sufficient training for clinicians and supervisors as well as ‘supervision of supervision’.

### Remote working

Because of the social-distancing measures brought in by the government to mitigate the spread of the Covid-19 virus, a significant proportion of the clinical work conducted in phases 1–3 will be completed remotely via the telephone or internet (Public Health England, 2020). This format of therapy delivery has encountered scrutiny for its questioned likeness to ‘real therapy’. More specific concerns have been raised about online facilitated therapy; for example, ‘the therapeutic alliance won’t be as strong online’ or ‘internet-based therapies won’t be as effective as face-to-face therapy’ (Thew, 2020; p. 4). However, by and large these concerns have been counterbalanced by evidence that suggests therapy of this format is effective, albeit different from therapy as ‘normal’. Above all, an undeniable strength of remote therapy, given social distancing, is its ability to enhance the availability and accessibility of psychological support (Thew, 2020). Similarly, telephone-delivered therapy has faced similar critiques, although research indicates that it remains able to provide the basis for a working alliance: a foundation of effective psychological support (Turner *et al*., 2018).

### Cultural adaptation

The workforce of NHS services is highly diverse both in nationality and ethnicity, with one in five (20.7%) from a Black, Asian and Minority Ethnic (BAME) background, and 13% from overseas nations. More specifically, it is estimated that around 29.7% of medical staff, who predominantly form the ‘frontline’, are ‘Asian’, whilst 4.6% were ‘Black’, 2.5% ‘Chinese’ and 3.2% ‘Mixed’. Within inner London boroughs such as Hackney (the borough of Homerton Hospital), BAME individuals make up a greater proportion of the NHS workforce (NHS Digital, 2020a). This is of utmost importance for mental health services to consider when delivering mental health interventions for frontline staff. Firstly, the cultural and ethnic diversity of staff needs to be acknowledged, with appreciation of the inequality and social injustice that exists between dominant and minority groups. Individuals from minority groups are more likely to experience discrimination, adversity and trauma: all risk factors for mental health difficulties (Levinson, 2011). Furthermore, according to the Intensive Care National Audit & Research Centre (ICNARC), BAME individuals made up 35% of approximately 2000 recorded Covid-19 cases who were hospitalised but form only 13% of the UK population (ICNARC, 2020). However, the reason for this disproportionate impact of the virus on BAME individuals is unknown. One possibility is that pre-existing health inequalities have resulted in poorer underlying health within some BAME individuals, therefore increasing their risk of developing severe and possibly life-threatening symptoms. This, which may exacerbate fear of infection, on top of the experiences from the frontline, may make BAME staff particularly vulnerable to mental health difficulties during the unfolding pandemic. As a result, HCPS is striving to acknowledge and overcome barriers that exist in the existing mental health system (especially during outreach and screening), to improve service access for BAME staff (Beck, 2019). Secondly, the interventions delivered to frontline staff of diverse backgrounds need to be adapted to take into account the differing conceptualisations of mental health and ‘recovery’ between cultures. Acknowledging this, the HCPS provisions delivered by its workforce will be closely monitored with regard to cultural competency as described by Sue *et al*. (2009). This will include case conceptualisation and supervision that recognises the therapist–client interactions impacted by cultural context as described by El-Leithy (2014).

### Evaluation plans

We intend to conduct a process evaluation covering the feasibility, acceptability and initial effectiveness of the service, drawing on the Medial Research Council (MRC) ‘evaluating complex interventions’ guidance (Moore *et al*., 2015). Standardised measures, commonly used in IAPT, will be used (where possible) across the phases. This consists of the Patient Health Questionnaire-9 (PHQ-9; Kroenke *et al*., 2001), the Generalised Anxiety Disorder-7 (GAD-7; Spitzer *et al*., 2006) and the Work and Social Adjustment Scale (WSAS; Mundt *et al*., 2002). The Traumatic Screening Questionnaire (TSQ; Brewin *et al*., 2002) will also be used across all phases to screen for and actively monitor trauma symptoms (NICE, 2018). The findings with regard to the pilot evaluation of the 20minCareSpace intervention offered during phase 2 will be included in the overall pilot evaluation being conducted by UCL (Scior and Clements, 2020).

## Concluding remarks

We hope to share our care pathway for frontline staff with other IAPT services who are planning similar approaches. In doing so, we hope to formulate a coordinated response to offering psychological support to hospital staff via pre-existing IAPT service infrastructures and workforces. We welcome requests to share our materials and invite other services to adopt our approach.

## Key practice points

(1) CBT has been delivered for frontline staff in disease outbreak settings based on the stepped-care IAPT model.
(2) These interventions would benefit from being phased to meet the needs of the frontline staff as they change depending on the stage of the outbreak.
(3) Interventions will require adaptation, given social distancing and due to the diversity of NHS frontline staff.

## Further reading

- For an example of a CBT-orientated service for frontline disease outbreak staff, see the papers evaluating the Ebola Psychological Support Service (Cole *et al.*, 2020; Waterman *et al.*, 2018; Waterman *et al.*, 2019).
- For expert guidance regarding the support required for frontline staff, see Greenberg and colleagues (2020).
- Thew (2020) provides an excellent review of research related to IAPT interventions delivered via the internet.
- El-Leithy (2014) explains the ways in which a CBT therapist can work in a manner that is sensitive to culture and diversity.

## References

[ref1] Albert, I. (2019). *Psychological Trauma Outreach, Screen and Support Service for London Terrorist Incidents October 2017–September 2019* Retrieved from: https://www.kcl.ac.uk/ioppn/depts/pm/research/imparts/Quick-links/Seminar-Slides/Seminar-15/06-albert-outreach-and-screen-slam.pdf

[ref2] Babin, B. J. , & Boles, J. S. (1996). The effects of perceived co-worker involvement and supervisor support on service provider role stress, performance and job satisfaction. Journal of Retailing, 72, 57–75. 10.1016/S0022-4359(96)90005-6

[ref3] Bailey, A. , & Heke, S. (2019). *Real World Trauma Therapy in a Changing Environment*. Workshop as part of ‘Making CBT Work in Challenging Times, London.

[ref4] Beck, A. (2019). Understanding Black and Minority Ethnic service users’ experience of racism as part of the assessment, formulation and treatment of mental health problems in cognitive behaviour therapy. the Cognitive Behaviour Therapist, 12. doi: 10.1017/S1754470X18000223

[ref5] Billings J. , Kember, T. , Greene, T. , Grey, N. , El-Leithy, S. , Lee, D. , … & Bloomfield, M. (2020). *Guidance for Planners of the Psychological Response to Stress Experienced by Hospital Staff Associated with COVID: Early Interventions* Retrieved from: https://232fe0d6-f8f4-43eb-bc5d-6aa50ee47dc5.filesusr.com/ugd/6b474f_5626bd1321da4138b1b43b78b8de2b20.pdf

[ref57] BMJ (2020). Covid-19: a physical and mental health epidemic - rapid response to Covid-19: a digital epidemic. *The BMJ, 368* Retrieved from: https://www.bmj.com/content/368/bmj.m764/rr

[ref56] Boyd, L. , Baker, E. , & Reilly, J. (2019). Impact of a progressive stepped care approach in an improving access to psychological therapies service: an observational study. PLoS ONE, 14. doi: 10.1371/journal.pone.0214715 PMC6456251 30964883

[ref6] Brewin, C. R. , Fuchkan, N. , Huntley, Z. , Robertonson, M. , Thompson, M. , Scragg, P. , d’Ardenne, P. , & Ehlers, A. (2010). Outreach and screening following the 2005 London bombings: usage and outcomes. Psychological Medicine, 40, 2049–2057. doi: 10.1017/S0033291710000206 20178677 PMC2964043

[ref7] Brewin, C. R. , Rose, S. , Andrews, B. , Green, J. , Tata, P. , McEvedy, C. , Turner, S. , & Foa, E. B. (2002). Brief screening instrument for post-traumatic stress disorder. British Journal of Psychiatry, 181, 158–162. 12151288 10.1017/s0007125000161896

[ref8] British Psychological Society (BPS) (2020). *Guidance for Psychological Professionals during the Covid-19 Pandemic* Retrieved from: https://www.bps.org.uk/sites/www.bps.org.uk/files/News/News%20-%20Files/Psychological%20needs%20of%20healthcare%20staff.pdf

[ref9] Brooks, S. , Amlot, R. , Rubin, G. J. , & Greenberg, N. (2020 ). Psychological resilience and post-traumatic growth in disaster-exposed organisations: overview of the literature. BMJ Military Health, 166, 52–56. doi: 10.1136/jramc-2017-000876 pmid:29420257 29420257

[ref10] Brooks, S. K. , Dunn, R. , Amlôt, R. , Rubin, G. J. , & Greenberg, N. (2016). A systematic, thematic review of social and occupational factors associated with psychological outcomes in healthcare employees during an infectious disease outbreak. Journal of Occupational and Environmental Medicine, 60, 248–257. doi: 10.1097/JOM.0000000000001235 29252922

[ref11] Brooks, S. K. , Dunn, R. , Sage, C. M. A. , Amlôt, R. , Greenberg, N. , & Rubin, J. (2015). Risk and resilience factors affecting the psychological wellbeing of individuals deployed in humanitarian relief roles after a disaster. Journal of Mental Health, 24, 385–413. doi: 10.3109/09638237.2015.1057334 26452755

[ref12] Cabinet Office (2020). *Guidance for schools, childcare providers, colleges and local authorities in England on maintaining educational provision* Retrieved from: https://www.gov.uk/government/publications/coronavirus-covid-19-maintaining-educational-provision/guidance-for-schools-colleges-and-local-authorities-on-maintaining-educational-provision

[ref13] Chen, Q. , Liang, M. , Li, Y. , Guo, J. , Fei, D. , Wang, L. … & Zhang, Z. (2020). Mental health care for medical staff in China during the COVID-19 outbreak. The Lancet Psychiatry, 7, 15–16. doi: 10.1016/S2215-0366(20)30078-X PMC7129426 32085839

[ref14] Clark, D. (2011). Implementing NICE guidelines for the psychological treatment of depression and anxiety disorders: the IAPT experience. International Review of Psychiatry, 23, 318–327. doi: 10.3109/09540261.2011.606803 22026487 PMC3212920

[ref15] Clark, D , Canvin, L. , Green, J. , Layard, R. , Pilling, S. , & Janecka, M. (2018). Transparency about the outcomes of mental health services (IAPT approach): an analysis of public data. The Lancet, 391, 679–686. doi: 10.1016/S0140-6736(17)32133-5 PMC5820411 29224931

[ref16] Cole, C. L. , Waterman, S. , Hunter, E. C. M. , Bell, V. , Greenberg, N. , Rubin, J. , & Beck, A. (2020). Effectiveness of small group cognitive behavioural therapy for anxiety and depression in Ebola treatment centre staff in Sierra Leone. International Review of Psychiatry. doi: 10.1080/09540261.2020.1750800 32301358

[ref17] Dean, W. , Talbot, S. , & Dean, A. (2019). Reframing clinician distress: moral injury not burnout. Federal Practitioner, 36, 400–402. 31571807 PMC6752815

[ref18] Delgadillo, J. , Asaria, M. , Ali, S. & Gilbody, S. (2016). On poverty, politics and psychology: the socioeconomic gradient of mental healthcare utilisation and outcomes. British Journal of Psychiatry, 209, 429–430. 26585097 10.1192/bjp.bp.115.171017

[ref19] El-Leithy, S. (2014). Working with diversity in CBT. In How to Become a More Effective CBT Therapist: Mastering Metacompetence in Clinical Practice, pp. 44–62. UK: Wiley-Blackwell.

[ref20] Flanagan, E. , Chadwick, R. , Goodrich, J. , Ford, C. , & Wickens, R. (2019). Reflection for all healthcare staff: a national evaluation of Schwartz Rounds. Journal of Interprofessional Care, 34, 140–142. doi: 10.1080/13561820.2019.1636008 31390225

[ref21] Flowers, S. , Bradfield, C. , Potter, R. , Waites, B. , Neal, A. , Simmons, J. , & Stott, N. (2018). ‘Taking care, giving care’ rounds: an intervention to support compassionate care amongst healthcare staff. *Clinical Psychology Forum* Retrieved from: https://www.researchgate.net/publication/323679757_‘Taking_care_giving_care’_rounds_An_intervention_to_support_compassionate_care_amongst_healthcare_staff

[ref22] Greenberg, N. , Docherty, M. , Gnanapragasam, S. , & Wessely, S. (2020). Managing mental health challenges faced by healthcare workers during covid-19 pandemic. BMJ, 368. doi: 10.1136/bmj.m1211 32217624

[ref23] Gerada, C. (2016). Healing doctors through groups: creating time to reflect together. British Journal of General Practice. doi: 10.3399/bjgp16X687469 PMC5033314 27688519

[ref24] Huang, D. , Yang, L. H. , & Pescosolido, B. A. (2019). Understanding the public’s profile of mental health literacy in China: a nationwide study. BMC Psychiatry, 19. doi: 10.1186/s12888-018-1980-8 PMC6332702 30642305

[ref25] Intensive Care National Audit and Research Centre (ICNARC) (2020). *ICNARC report on COVID-19 in critical care 10 April 2020* Retrieved from: https://www.icnarc.org/DataServices/Attachments/Download/c31dd38d-d77b-ea11-9124-00505601089b

[ref26] Jackson, H. (2018). Retaining and valuing newly qualified nursing staff: evaluation of a peer support group. Mental Health Practice. doi: 10.7748/mhp.2018.e1241

[ref27] Kroenke, K. , Spitzer, R. L. , & Williams, J. B. (2001). The PHQ-9: validity of a brief depression severity measure. Journal of General Internal Medicine, 16, 606–613. 11556941 10.1046/j.1525-1497.2001.016009606.x PMC1495268

[ref28] Lai, J. , Ma, S. , & Wang, Y. (2020). Factors associated with mental health outcomes among health care workers exposed to Coronavirus disease 2019. JAMA Network Open, 3. doi: 10.1001/jamanetworkopen.2020.3976 PMC7090843 32202646

[ref29] Levinson, M. (2011). *The Civic Empowerment Gap: Defining the Problem and Locating Solutions* Retrieved from: https://dash.harvard.edu/bitstream/handle/1/8454069/Levinson%20The%20Civic%20Empowerment%20Gap.pdf

[ref30] Maslow, A. H. (1943). A theory of human motivation. Psychological Review, 50, 370–396.

[ref31] Makwana, N. (2019). Disaster and its impact on mental health: a narrative review. Journal of Family Medicine and Primary Care, 8, 3090–3095. doi: 10.4103/jfmpc.jfmpc_893_19 PMC6857396 31742125

[ref32] McKinley, N. , McCain, R. S. , & Convie, L. (2020). Resilience, burnout and coping mechanisms in UK doctors: a cross-sectional study. BMJ Open, 10. doi: 10.1136/bmjopen-2019-03176 PMC7045750 31988223

[ref33] Moore, G. F. , Audrey, S. , Barker, M. , Bond, L. , Bonell, C. , Hardeman, W. , … & Baird, J. (2015). Process evaluation of complex interventions: Medical Research Council guidance. The BMJ, 350. doi: 10.1136/bmj.h1258 PMC4366184 25791983

[ref34] Mundt, J. C. , Marks, I. M. , Shear, M. K. , & Greist, J. M. (2002). The Work and Social Adjustment Scale: a simple measure of impairment in functioning. The British Journal of Psychiatry, 180, 461–464. 11983645 10.1192/bjp.180.5.461

[ref35] National Collaborating Centre for Mental Health (2019). *The Improving Access to Psychological Therapies Manual* Retrieved from: https://www.england.nhs.uk/wp-content/uploads/2019/12/iapt-manual-v3.pdf

[ref36] NHS Digital (2020a). *NHS Workforce – Ethnicity Facts and Figures* Retrieved from: https://www.ethnicity-facts-figures.service.gov.uk/workforce-and-business/workforce-diversity/nhs-workforce/latest

[ref37] NHS Digital (2020b). *Psychological Therapies: Reports on the Use of IAPT Services, England – January 2020* Retrieved from: https://digital.nhs.uk/data-and-information/publications/statistical/psychological-therapies-report-on-the-use-of-iapt-services/january-2020-final-including-reports-on-the-iapt-pilots

[ref38] NICE (2011). *Common mental health disorders: identification and pathways to care. Clinical Guideline 123* Retrieved from: www.nice.org.uk/guidance/cg123

[ref39] NICE (2018). *Post-traumatic stress disorder – NICE guideline* [NG116]. Retrieved from: https://www.nice.org.uk/guidance/ng116

[ref40] Perlis, R. H. (2020). Exercising heart and head in managing Coronavirus disease 2019 in Wuhan. JAMA Network Open. doi: 10.1001/jamanetworkopen.2020.4006 32202641

[ref41] Public Health England (2020). *Guidance on Social Distancing for Everyone in the UK* Retrieved from: https://www.gov.uk/government/publications/covid-19-guidance-on-social-distancing-and-for-vulnerable-people/guidance-on-social-distancing-for-everyone-in-the-uk-and-protecting-older-people-and-vulnerable-adults

[ref42] Raven, J. , Wurie, H. & Witter, S. (2018). Health workers’ experiences of coping with the Ebola epidemic in Sierra Leone’s health system: a qualitative study. BMC Health Service Research, 18. doi: 10.1186/s12913-018-3072-3 PMC5887191 29622025

[ref43] Scior, K. , & Clements, H. (2020). Proposed Pilot and Formative Evaluation of 20minCareSpace. Unpublished manuscript.

[ref44] Spitzer, R. L. , Kroenke, K. , Williams, J. B. , & Lowe, B. (2006). A brief measure for assessing generalized anxiety disorder: the GAD-7. Archives of Internal Medicine, 166, 1092–1097. 16717171 10.1001/archinte.166.10.1092

[ref45] Sue, S. , Zane, N. , Nagayama Hall, G. C. , & Berger, L. K. (2009). The case for cultural competency in psychotherapeutic interventions. Annual Review of Psychology, 60, 525–548. 10.1146/annurev.psych.60.110707.163651 PMC2793275 18729724

[ref55] Sull, A. , Harland, N. , & Moore, A. (2015). Resilience of health-care workers in the UK; a cross-sectional survey. Journal of Occupational Medicine and Toxicology, 10. doi: 10.1186/s12995-015-0061-x. eCollection 2015 PMC4449529 26029246

[ref46] Sweeney, A. , & Taggart, D. (2018). (Mis)understanding trauma-informed approaches in mental health. Journal of Mental Health, 27. doi: 10.1080/09638237.2018.1520973 30345848

[ref47] Taylor, C. , Xyrichis, A. , Leamy, M. C. , Reynolds, E. , & Maben, J. (2018). Can Schwartz Center Rounds support healthcare staff with emotional challenges at work, and how do they compare with other interventions aimed at providing similar support? A systematic review and scoping reviews. BMJ Open, 8. doi: 10.1136/bmjopen-2018-024254. PMC6196967 30341142

[ref48] Thew, G. R. (2020). IAPT and the internet: the current and future role of therapist-guided internet interventions within routine care settings. the Cognitive Behaviour Therapist, 13, 1–11. doi: 10.1017/S1754470X20000033 PMC8442601 34567240

[ref49] Turner, J. , Brown, J. C. , & Carpenter, D. T. (2018). Telephone-based CBT and the therapeutic relationship: the views and experiences of IAPT practitioners in a low-intensity service. Journal of Psychaitric and Mental Health Nursing, 25, 285–296. doi: 10.1111/jpm.12440 29117458

[ref50] Unadkat, S. & Farquhar, M. (2020). *Doctors’ Wellbeing: Self-Care During the Covid-19 Pandemic* Retrieved from: https://blogs.bmj.com/bmj/2020/03/16/self-care-during-the-covid-19-pandemic/ 10.1136/bmj.m1150 32209559

[ref51] Waterman, S. , Hunter, E. C. M. , Cole, C. L. , Evans, L. J. , Greenberg, N. , Rubin, G. J. , & Beck, A. (2018). Training peers to treat Ebola centre workers with anxiety and depression in Sierra Leone. International Journal of Psychiatry, 64, 156–165. doi: 10.1177/0020764017752021 29432085

[ref52] Waterman, S. , Cole, C. L. , Greenberg, N. , Rubin, G. J. , & Beck, A. (2019). A qualitative study assessing the feasibility of implementing a group cognitive-behavioural therapy-based intervention in Sierra Leone. BJPsych International, 16, 31–34. doi: 10.1192/bji.2018.7 31144684 PMC6520535

[ref53] WHO (2020). *Mental Health and Psychosocial Considerations During COVID-19 Outbreak* Retrieved from: https://www.who.int/docs/default-source/coronaviruse/mental-health-considerations.pdf?sfvrsn=6d3578af_8

[ref54] Wu, P. , Fang, Y. , Guan, Z. , Fan, B. , Kong, J. , Yao, Z. , … & Hoven, C. W. (2009). The psychological impact of the SARS epidemic on hospital employees in China: exposure, risk perception, and altruistic acceptance of risk. Canadian Journal of Psychiatry, 54, 302–311. doi: 10.1177/070674370905400504 19497162 PMC3780353

